# Electron‐withdrawing inductive effects enhanced strategy for protein thiol sensing and blocking agent design

**DOI:** 10.1002/smo.20230022

**Published:** 2024-02-14

**Authors:** Liangwei Zhang, Shudi Liu, Xia Zhang, Jinyu Sun, Lingxin Chen

**Affiliations:** ^1^ CAS Key Laboratory of Coastal Environmental Processes and Ecological Remediation Yantai Institute of Coastal Zone Research Chinese Academy of Sciences Yantai China; ^2^ College of Chemistry and Chemical Engineering Yantai University Yantai China; ^3^ Department of Biochemistry and Molecular Biology Binzhou Medical University Yantai China; ^4^ Institute of Medical Research Northwestern Polytechnical University Xi'an China; ^5^ College of Chemistry and Chemical Engineering Shaoxing University Shaoxing China

**Keywords:** bio‐thiol, fluorescent sensing, Parkinson's disease, protein labeling, sulfone

## Abstract

It is a great challenge to discover novel chemical reactions suitable for biological analysis in a living system. The development of novel protein thiol blocking agents is a crucial need for exploring protein thiol functions in protein refolding, signal transduction, and redox regulation. We are always keen on seeking novel chemical reactions applied to endogenous biological macromolecules or protein thiol sensing, blocking, and labeling. In the present work, we have successfully developed a novel agent to block protein thiol by enhanced electron‐withdrawing inductive effects. This sensing and blocking process was detailedly monitored by UV‐*vis*, fluorescent spectra, and SDS‐Page gel separation. The spectral studies demonstrated that the agent could react ultrafastly with thiol within seconds at μM level. Furthermore, fluorescent imaging in cells and in vivo was further used for the validation of its ability to sensing and blocking thiol, providing evidence of downregulated protein thiols in Parkinson's disease. The enhanced electron‐withdrawing inductive effect strategy in this work may provide a general guideline for designing protein thiol agent.

## Graphical abstract

We have successfully developed a novel design strategy for protein thiols blocking and sensing agent by tuning electron‐withdrawing inductive effects to improve its performances and applied it in fluorescent sensing and labeling in living cells and in vivo.

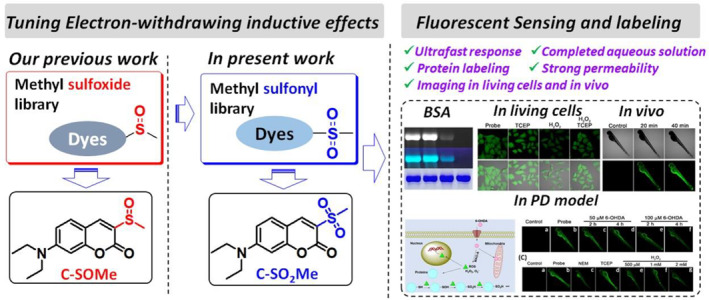

## 1 INTRODUCTION

Numerous disease occurrence are keeping close relationships with improper post‐translational modifications caused by oxidative stress derived from external changes, such as physical factors and environmental contaminants.^[1]^ Developing novel chemical toolbox for exploring post‐translational modifications of biological molecules is of vital importance to understand the physiological and pathological processes in the living system.[^2^, ^3^, ^4^, ^5^, ^6^, ^7^] Thereinto, exploring effective methods applied in the living systems to unveil the biological functions of macromolecules in redox regulation is meaningful. One of the key objectives is developing the proper chemical toolbox for bio‐thiols. As one kind of antioxidants, the bio‐thiols, including small molecule thiols and thiol residues in proteins, play vital roles in maintaining redox homeostasis to defense against oxidative stress. The consumption of the bio‐thiols would form a series of different oxidative intermediates, such as disulfide, sulfenic acid, sulfinic acid, and sulfonic acid.[^8^, ^9^, ^10^] Therefore, discovering new chemical reactions that occurred under mild conditions and can be applied in living systems to monitor cellular events is the important breakthrough. Up to now, a large number of organic small molecules have been developed by decorating functional recognition groups onto various fluorophores and acted as fluorescent probes for this purpose.[^11^, ^12^, ^13^, ^14^, ^15^, ^16^, ^17^, ^18^, ^19^] In combination with fluorescent microscopy, small organic fluorescent molecules exhibit indispensable advantages for insight into biological events, especially in the aspects of non‐destructive imaging and visualization monitoring.[^20^, ^21^, ^22^, ^23^, ^24^, ^25^, ^26^, ^27^] However, the crucial factor in probe design is to discover chemical reactions with high specificity toward the target and reasonably tune the optical performances.

**FIGURE 1:**
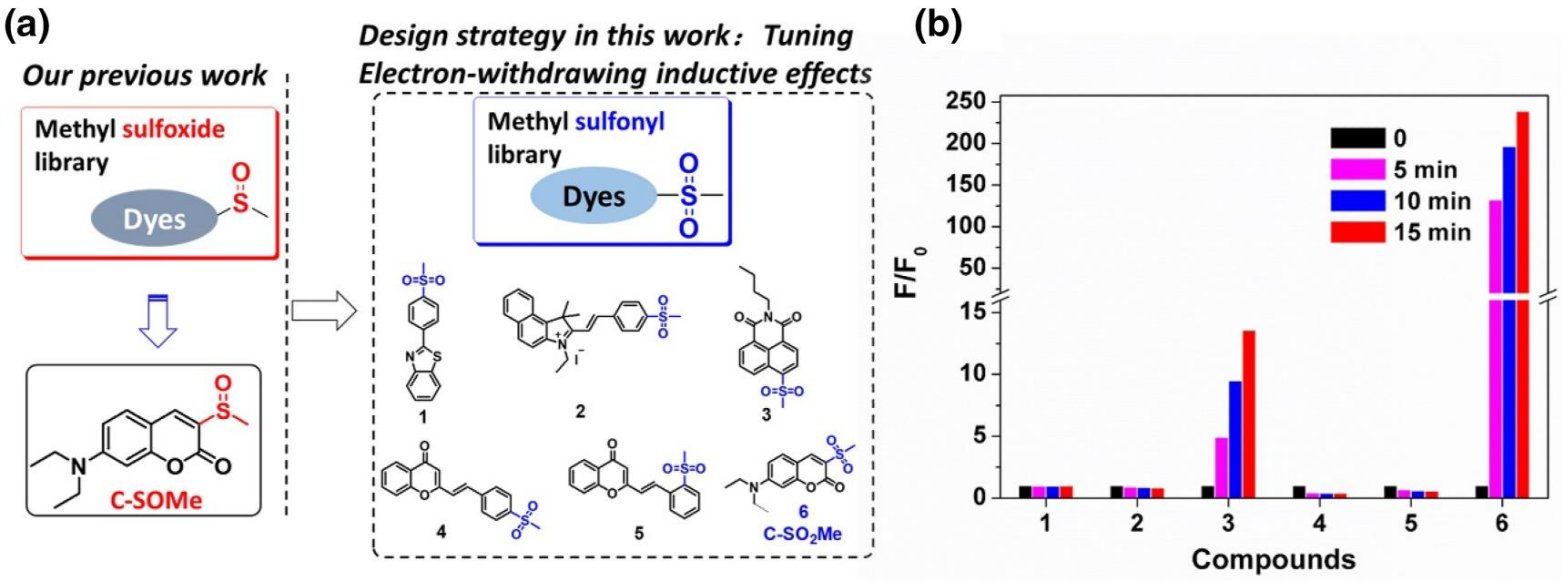
(a) Improved fluorescent probe design strategy; (b) Compounds screening by incubation of the probe (10 μM) with GSH (10 μM) for different times at room temperature. (*λ*_ex_ = 370 nm, *λ*_em_ = 508 nm, in 10 mM PBS buffer, pH = 7.40).

In recent years, we are always trying to seek novel chemical reactions to unveil the biological functions of macromolecules in redox regulation. We have designed a series of small organic molecule probes for monitoring the activity of biological macromolecules based on their enzyme catalytic characteristics, such as the first thioredoxin reductase probe TRFS‐green and the methionine sulfoxide reductase probes (Msr‐blue, Msr‐Ratio and Msr‐TFMCM).[^28^, ^29^, ^30^, ^31^] Based on our previous studies, we have constructed a methyl sulfoxide library (Figure 1a), showing that the methyl sulfoxide group is a good leaving group when it met a nucleophilic reagent in a push‐pull conjugate system, *viz.* A molecule containing electron withdrawing group (EWG) − π system − electron donating group (EDG).^[32]^ Inspired by this, we hypothesized that sulfone might have a stronger leaving ability than the sulfoxide group. Having deeply investigated the topic, we found that only several sulfone‐based organic compounds were developed for labeling or sensing thiols. However, these compounds suffered from different disadvantages, mainly including a large proportion of organic solvents, low reaction activity, and poor optical properties. Xian et al. developed methylsulfonyl benzothiazole for protein thiol blocking, which required more than 20 min to complete this process in a mixed aqueous solution containing a large proportion of organic solvents (THF/PBS, 1:2).^[33]^ Fang and Kong's labs synthesized naphthalimide‐based methylsulfonyl compounds for labeling or sensing biothiols. However, the reaction time of this kind of methylsulfonyl compounds with thiols needs a longer response time (more than half an hour) at the level of mmol/L.[^34^, ^35^] Therefore, it is a great challenge to elevate the response rate and optical property by tuning the leaving ability of sulfone group in a push‐pull conjugate system.

To solve the problems, we selected several typical fluorophores containing π‐conjugated system uniformly furnished with methylsulfonyl groups to construct a small library, including phenylbenzothiazole, cyanine, naphthalimide, chromone, and coumarin (Figure 1a). All the compounds were simply synthesized within two‐steps and fully characterized in supporting information. Screening by spectral studies (Figure 1b), we found the type of conjugated system in coumarin skeleton 6 (C‐SO_2_Me) showing ultrahigh react activity. The probe C‐SO_2_Me could quickly react with low concentration of biological thiols in a complete aqueous solution, showing the turn on fluorescent enhancement signals and high selectivity undisturbed by other endogenous biomolecules. To validate the practical sensing and blocking ability of the probe in a living system, we have constructed different oxidative stress models to observe the thiol changes, including a direct oxidative model and neurodegenerative disease model. The fluorescence imaging results in living cells and in vivo sufficiently demonstrated that the probe is a powerful tool for revealing the roles of biological thiols in redox regulation by monitoring their fluctuations.

## 2 RESULTS AND DISCUSSION

**FIGURE 2:**
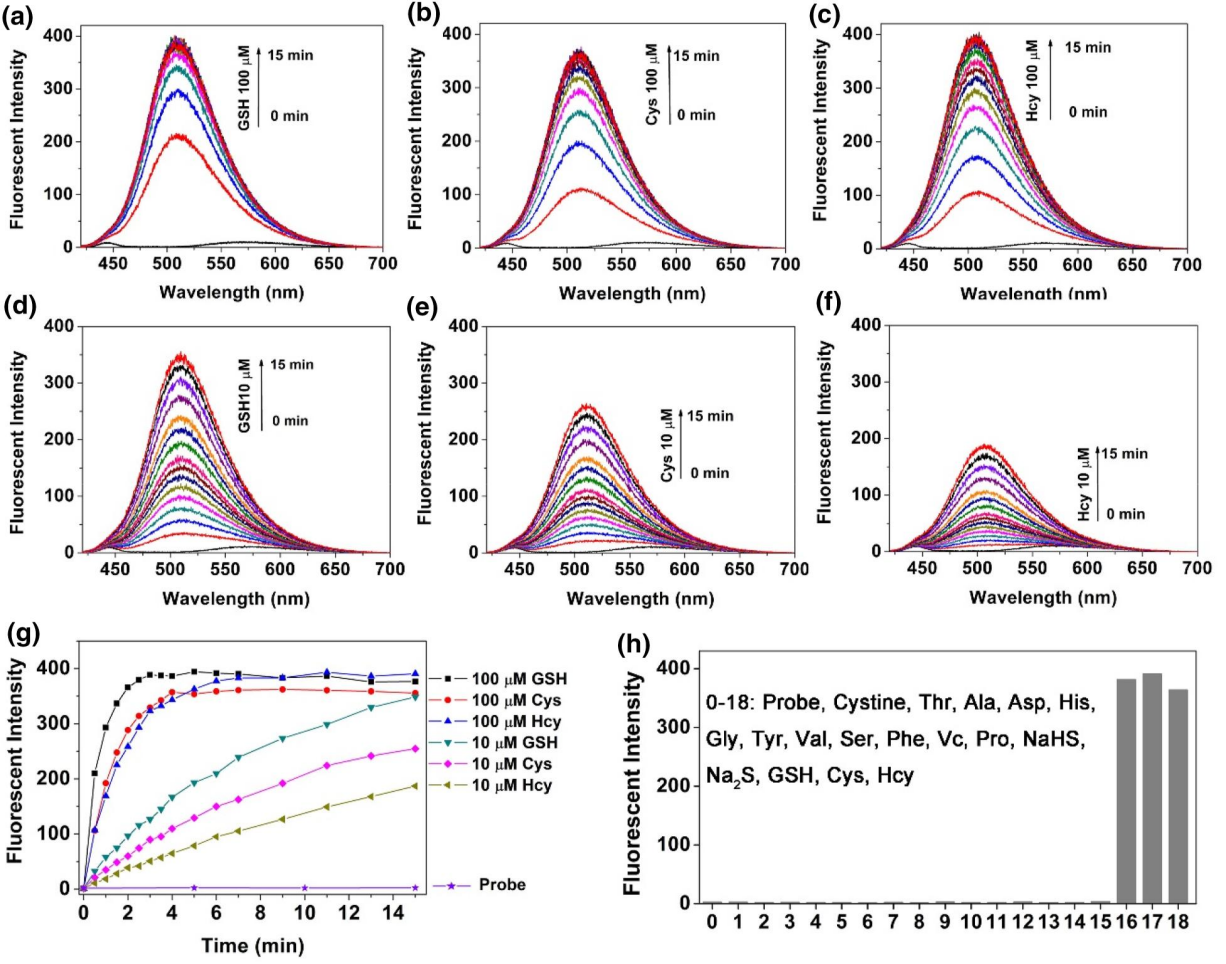
Fluorescent spectral studies of probe C‐SO_2_Me (10 μM). Fluorescent responses of C‐SO_2_Me with GSH, Cys, and Hcy (a–c, 100 μM; d–f, 10 μM); (g) Time‐ and dose‐dependent fluorescent responses of C‐SO_2_Me with thiols at 508 nm according to a‐f; (h) Fluorescent responses of C‐SO_2_Me (10 μM) toward various species (100 μM).

With these methylsulfonyl compounds in hand, we firstly investigated their response ability toward biological thiols. After screening via spectral studies, the fluorescent probe C‐SO_2_Me was picked up from the library for detecting small molecule biological thiols, showing high sensitivity and selectivity. In Figure 2, we have detailedly studied the dose‐ and time‐dependent fluorescent response of the probe C‐SO_2_Me with the bio‐thiols, including GSH, Cys, and Hcy. The results confirmed that the probe C‐SO_2_Me could quickly react with thiols and reach up to saturation within 120 s at μM level in completed aqueous solution (10 mM PBS buffer, pH = 7.40). The changes in fluorescent emission may be attributed to the intramolecular charge transfer (ICT) process that the methylsulfonyl moiety (electron withdrawing) has been substituted and transformed into the corresponding sulfide (electron donating), and the reaction mechanism was confirmed by HRMS spectra (Figure S1). The response rate order obtained from Figure 2g is that GSH ﹥ Cys ﹥ Hcy, which is accordance with our previous results of the probe C‐SOMe and may relate to their different p*K*_a_ values.^[32]^ The fluorescent response performances were greatly improved compared with the reported work.[^33^, ^34^, ^35^] Then, the selectivity experiments were investigated in Figure 2h; only biological thiols could cause a remarkably fluorescence enhancement of more than 300‐fold and other amino acids and endogenous reactive species did not induce a fluorescence response. All these solid results confirmed that the probe C‐SO_2_Me could react ultrafastly and specifically with biological thiols with high sensitivity and selectivity in a completed aqueous solution.

**FIGURE 3:**
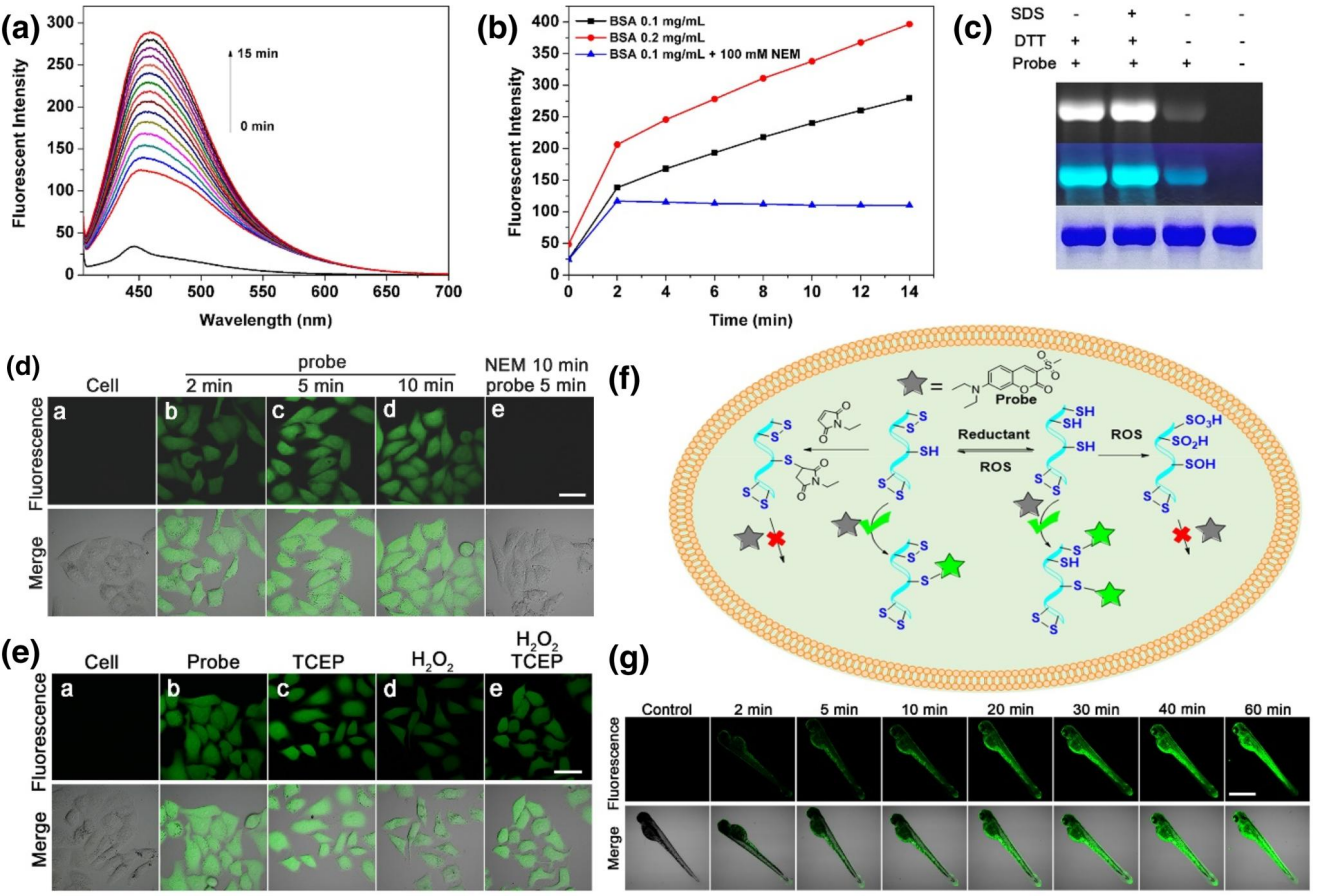
(a) Fluorescent changes of C‐SO_2_Me with BSA (0.1 mg/mL) as time increased; (b) Time‐ and dose‐dependent fluorescent response of C‐SO_2_Me (10 μM) toward BSA; (c) Different BSA samples with C‐SO_2_Me on a SDS‐Page gel. (d) Time‐dependent fluorescent changes of C‐SO_2_Me (10 μM) in cells (a–d). The cells were pretreated with NEM (100 μM) for 10 min and then incubated with the probe (10 μM) (e); (e) Fluorescent fluctuations of C‐SO_2_Me in living cells. (a) The cells. (b) The cells were incubated with the probe (10 μM) for 5 min. (c) The cells were pretreated with 500 μM TCEP for 30 min and then incubated with the probe (10 μM). (d) The cells were pretreated with 500 μM H_2_O_2_ for 30 min and then incubated with the probe (10 μM). (e) The cells were sequentially pretreated with 500 μM H_2_O_2_ (30 min) and TCEP (30 min), then incubated with the probe (10 μM); (f) Diagram of probe labeling protein under different cases; (g) Time‐dependent fluorescent changes files of probe (10 μM) in living zebra fish. Scale bar: 40 μm for cell and 400 μm for zebra fish.

Having confirmed its brilliant performances of sensitivity and selectivity toward thiols in completed aqueous solution, we try to check its practical applications in sensing and blocking biological thiols in a living system. Before this, we selected BSA as an external contrast model to check the ability of the probe C‐SO_2_Me to react with sulfhydryl group in protein. In Figures 3a,b, it exhibited excellent time‐ and dose‐dependent fluorescent enhancement. The thiol‐blocking agent N‐ethylmaleimide (NEM) could suppress the enhancement, which indirectly indicates the selectivity of the probe toward thiols. Then, it was used to label BSA samples with different handling methods and separated on a SDS‐Page gel (Figure 3c). A remarkable fluorescent enhancement was observed when the dithiols in BSA were reduced by dithiothreitol (DTT), confirming that the probe has strong labeling ability of proteins containing sulfhydryl group. Interestingly, whenever BSA was treated with or without SDS (Sodium dodecyl sulfate, a protein denaturation reagent), the results showed very slight fluorescent difference, indicating that our developed agent C‐SO_2_Me has great potential application in labeling thiols in protein.

Before imaging in cells, we have carried out cytotoxicity assays in HepG2 cells to assess biocompatibility (Figure S2). In Figure 3d, we investigated the time‐dependent response of the probe C‐SO_2_Me with thiols in cells. Strong green fluorescence signals were observed after incubation with the probe within 5 min, and then it remained stable as the time increased, proving that the probe could penetrate into cells and react with thiols quickly and the probe has high optical stability, which is a very vital factor for imaging analysis. To validate the specificity with thiols, the thiol‐blocking agent NEM was also used for scavenging them. As shown in Figure 3d, there were almost no fluorescence signals in the cells when pretreated with NEM (100 μM). In addition, we investigated the response ability of the probe to exogenous thiols, including GSH, Cys, and Hcy. In Figure S3, the fluorescence signal enhancement order remains in accordance with that in aqueous solution, *viz.* GSH ﹥ Cys ﹥ Hcy. All these results suggested that the probe was an excellent agent that could quickly reacts with biological thiols in moderated conditions and act as a useful tool for sensing and blocking analysis.

The biological thiols play key roles in regulating redox balance through variation of their different sulfide intermediates, including sulfhydryl, disulfide, sulfenic acid, sulfinic acid, sulfonic acid, and so on.[^8^, ^10^] Abnormal expression levels of biological thiols occur in the pathological processes such as cancer and neurodegenerative disease.[^36^, ^37^, ^38^] Therefore, real‐time monitoring of biological thiol fluctuation is of great importance for elucidating their functions. Relative upregulated and downregulated experiments for an oxidative stress model in living cells were carried out to observe the thiol changes. On the one hand, the fluorescent signal was enhanced when the cells were pretreated with tris(2‐carboxyethyl)‐phosphine (TCEP), known as a reducing agent to cleave disulfide in a biological system, before incubation with the probe (Figure 3Ec). This can be attributed to the major reason that the disulfide in protein were opened to enrich the available sulfhydryl in this targeting process.^[39]^ On the other hand, oxidants could consume sulfhydryl and transfer it to other oxidative intermediates.[^8^, ^10^] As shown in Figure 3Ed, exogenous hydrogen peroxide (H_2_O_2_) was aforehand incubated with the cells and resulted in the significantly suppressed fluorescence signals, showing very weak green fluorescence. As the concentrations of H_2_O_2_ increased, the fluorescence intensity weakened gradually (Figure S4). Inversely, compared with the fluorescence in Figure 3Ed, the fluorescent signals in cells could be remarkably recovered when the cells sequentially incubated with H_2_O_2_ and TCEP (Figure 3Ee). However, the fluorescent signals were obviously weaker than that in Figure 3Ec, suggesting that the content of thiols couldn't reach up to previous level even though using the TCEP to destruct formed disulfide. This may be ascribed to the reason that part of the sulfhydryl group transformed into other intermediates rather than disulfide during the oxidative process. All the above response mechanisms of fluorescent signals in cells can be detailedly summarized in Figure 3f. After validation of its sensing and labeling performance in vitro, we further investigated the conjugation ability of the probe C‐SO_2_Me with biothiols in vivo. In Figure 3g, green fluorescence was observed and enhanced sharply as the incubation time of the probe increased in living zebra fish. All these solid results firmly confirmed that the probe is a vital sensing and labeling tool for monitoring the fluctuations of biological thiols in redox systems.

**FIGURE 4:**
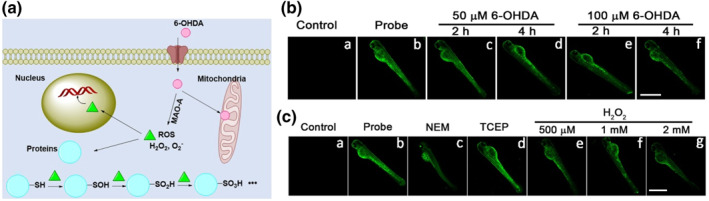
(a) The possible mechanism of 6‐OHDA‐induced PD model and its effects on proteins; (b) Fluorescent imaging of probe in PD model: (a) The zebra fish. (b) The zebra fish were incubated with the probe (10 μM) for 20 min. The zebra fish were pretreated with 6‐OHDA (50 μM) for 2 h (c) or 4h (d) and then incubated with the probe (10 μM). The zebra fish were pretreated with 6‐OHDA (100 μM) for 2h (e) or 4h (f) and then incubated with probe (10 μM). (c) Monitoring biological thiol fluctuations in living zebra fish. (a) The zebra fish. (b) The zebra fish were incubated with the probe (10 μM) for 20 min. (c) The zebra fish were pretreated with NEM (100 μM) for 30 min and then incubated with the probe (10 μM). (d) The zebra fish were pretreated with TCEP (100 μM) for 30 min and then incubated with the probe (10 μM). (e–f) The zebra fish were pretreated with H_2_O_2_ for 30 min and then incubated with the probe (10 μM). Scale bar: 400 μm.

Aging and neurodegenerative diseases are closely related to oxidative stress.^[40]^ Parkinson's disease (PD), as one of the typical neurodegenerative diseases, was selected for studying the biological thiol fluctuations. The PD model was commonly constructed by using 6‐hydroxydopamine (6‐OHDA) which was known as neurotoxin yielding potentially toxic products and reactive oxygen species (Figure 4a); however, it is not clear.^[41]^ Therefore, we intend to use the probe to monitor biothiol fluctuation, revealing the roles of bithiols in the PD model. In Figure 4b, the fluorescent signals in zebra fish showed a time‐ and dose‐dependent damping. The fluorescence signal gradually dimmed as the incubation time and concentrations of 6‐OHDA increased. To further confirm the fluorescent decay caused by 6‐OHDA induced oxidative stress,^[42]^ the probe was applied in monitoring biological thiols fluctuations in different upregulated and downregulated zebra fish models. As shown in Figure 4c, the fluorescent signals were greatly inhibited when zebra fish was pretreated with NEM. Oppositely, the fluorescence with a certain enhancement was observed when the zebra fish was pretreated with TCEP. Under oxidative stress induced by exogenous addition of H_2_O_2_, fluorescent signals decay gradually as their concentration increases (Figure 4c). All the imaging in zebra fish suggests that the probe has strong ability to react with biological thiols in vivo. The fluorescent changes indirectly indicated that biological thiol would be downregulated in Parkinson's disease.

## 3 CONCLUSION

In summary, we have successfully synthesized a series of compounds containing methylsulfonyl groups in different pull–push conjugated fluorophore systems to improve the reaction ability with thiols. The electron‐withdrawing inductive effects were productive for discovering novel sensing and protein labeling strategies. We have also picked up the probe C‐SO_2_Me to monitor the fluctuations of biological thiols with high reactivity and selectivity in completed aqueous solutions. The imaging in living cells and in vivo demonstrated that the probe has excellent biological compatibility and could quickly sense and conjugate endogenous small molecule thiols and protein thiols. The direct oxidative model and neurodegenerative disease model further validate the practical sensing and blocking ability of the probe. Our studies may enlighten the readers to develop novel agents and strategies for sensing and protein labeling.

## CONFLICT OF INTEREST STATEMENT

The authors declare no conflicts of interest.

## ETHICS STATEMENT

No animal or human experiments were involved in this study.

## Supporting information

Supplementary information S1

## Data Availability

The data that support the findings of this study are available from the corresponding authors upon reasonable request.
