# Supplementary material for: Electron‐withdrawing inductive effects enhanced strategy for protein thiol sensing and blocking agent design
Source: Smart Mol. 2024 Feb 14;2(1):e20230022. doi: 10.1002/smo.20230022 (PMC12118245; doi:10.1002/smo.20230022)
Supplement: Supplementary file 1 — Supplementary information S1 [file SMO2-2-e20230022-s001.docx]

((Supporting Information can be included here using this template))

Supporting Information

Electron-withdrawing inductive effects enhanced strategy for protein thiol sensing and blocking agent design

Liangwei Zhang,^a^ Shudi Liu,*^b^ Xia Zhang,^c^ Jinyu Sun^d^ and Lingxin Chen*^a,e^

*^a^* CAS Key Laboratory of Coastal Environmental Processes and Ecological Remediation, Yantai Institute of Coastal Zone Research, Chinese Academy of Sciences, Yantai 264003, China.

*^b^* College of Chemistry and Chemical Engineering, Yantai University, Yantai 264005, China

*^c^* Department of Biochemistry and Molecular Biology, Binzhou Medical University, Yantai 264003, China

*^d^* Institute of Medical Research, Northwestern Polytechnical University, Xi’an, 710072, China

*^e^* College of Chemistry and Chemical Engineering, Shaoxing University, Shaoxing 312000, China

* E-mail: liushd2018@163.com (S. Liu); lxchen@yic.ac.cn (L. Chen).

**E-mail:** liushd2018@163.com; lxchen@yic.ac.cn

**1. Materials and instruments**

All reagents were purchased from commercial supplies without further purification. NMR spectra were recorded on Bruker 500 MHz instruments. MS spectrum was performed on Trace DSQ GC-MS spectrometer (Thermo). Live cell imaging was carried out on inverted fluorescent microscope (Fluo View FV1000). Fluorescence studies were recorded on an Agilent Cary Eclipse Fluorescence Spectrophotometer. The gel imaging was performed by Tanon 2500 Gel Imaging System.

**2. Fluorescent imaging in living cell and zebra fish.**

Hep G2 cells (4x10^5^) were cultured in Dulbecco’s Modified Eagle Medium (DMEM) in plates and allowed to grow overnight. Then C-SO_2_Me (10 μM) was added to the plate and continued culture for certain time. With difference, the intracellular thiols were blocked by addition of NEM 30 minutes ahead the addition of the probe. Oxidative stress model of cells was pre-treated with different concentrations of H_2_O_2_ for 30 min, and then was incubated with probe C-SO_2_Me (10 μM) for 5 min. 3-days zebra fish was treated with 6-OHDA for different time and then treated with probe for 20 min. The cells and fish were visualized and photographed on inverted fluorescent microscope (Fluo View FV1000).

**3. BSA labeling**

BSA (1 mg/mL) was dissolved in PBS (10 mM, pH = 7.4) and then handled with different process. (1) BSA was dealt with 10% SDS for 30 min and reduced by 500 µM (DTT) for 30 min. The precipitate was collected by addition of cold acetone (v/v, 1:2) kept at −20 °C for 30 min and centrifuging at 4 °C for 10 min. (2) BSA was directly reduced by 500 µM (DTT) for 30 min and the precipitate was collected by addition of cold acetone (v/v, 1:2) kept at −20 °C for 30 min and centrifuging at 4 °C for 10 min. In the above process, excess DTT was removed by acetone precipitation. (3) The above two samples and BSA were dissolved in PBS and labeled with C-SOMe (10 μM) at 37 °C, and then the protein samples were separated on a SDS-Page gel. The same gel was also stained by Coomassie brilliant blue (CBB) after the fluorescent image was obtained.

**
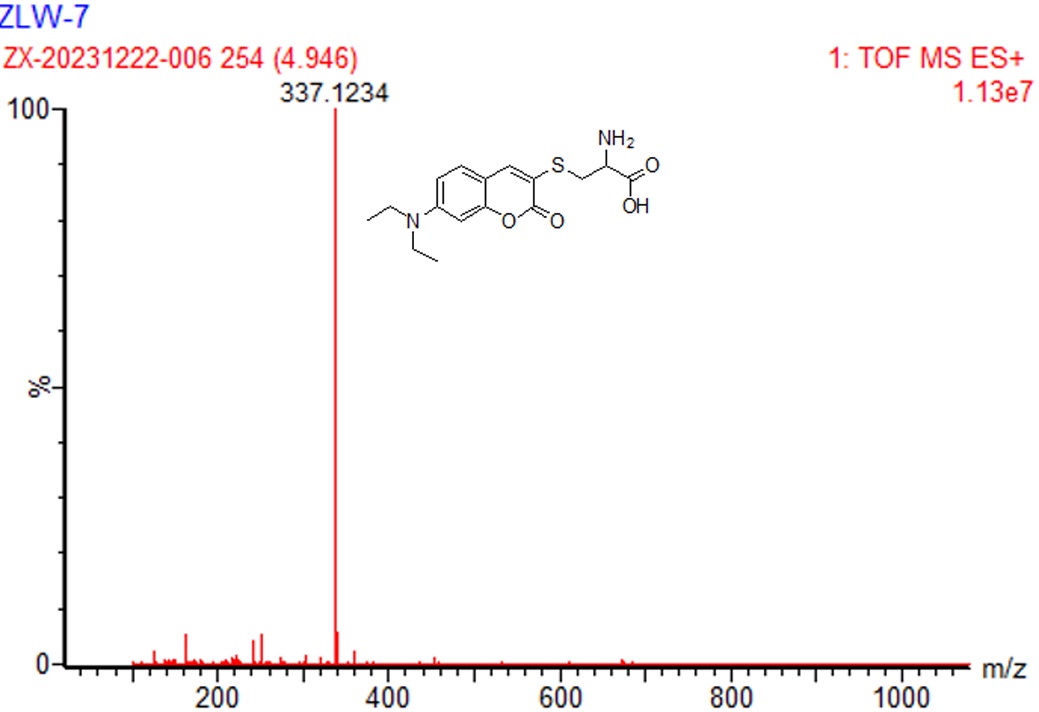
**

**Figure S1** The HRMS spetrum of the probe reacted with Cys.

**

**

**Figure S2** Cytotoxicity assays of probe C-SO_2_Me in HepG 2 cells for 12h.

**
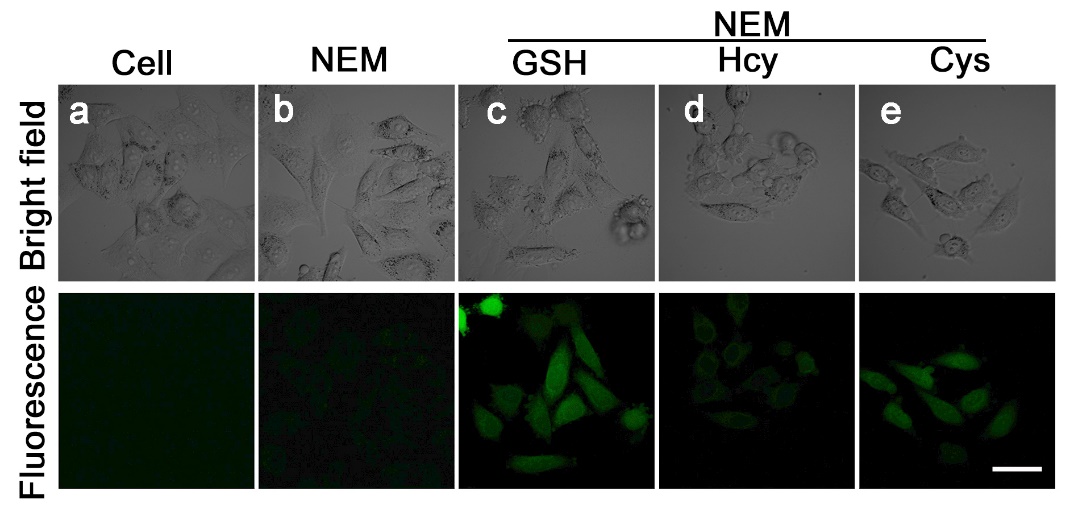
**

**Figure S3** Fluorescent response of C-SO_2_Me to exogenous thiols. (a) The cells; (b) The cells pre-treated with NEM (100 μM) for 30 min and then incubated with probe (10 μM) for 5 min; The cells pre-treated with NEM (100 μM) for 30 min and then incubated with 100 μM of GSH (c), Hcy (d), and Cys (e) for 30 min, and then incubated with probe (10 μM) for 5 min. Scale bar: 40 μm.

**
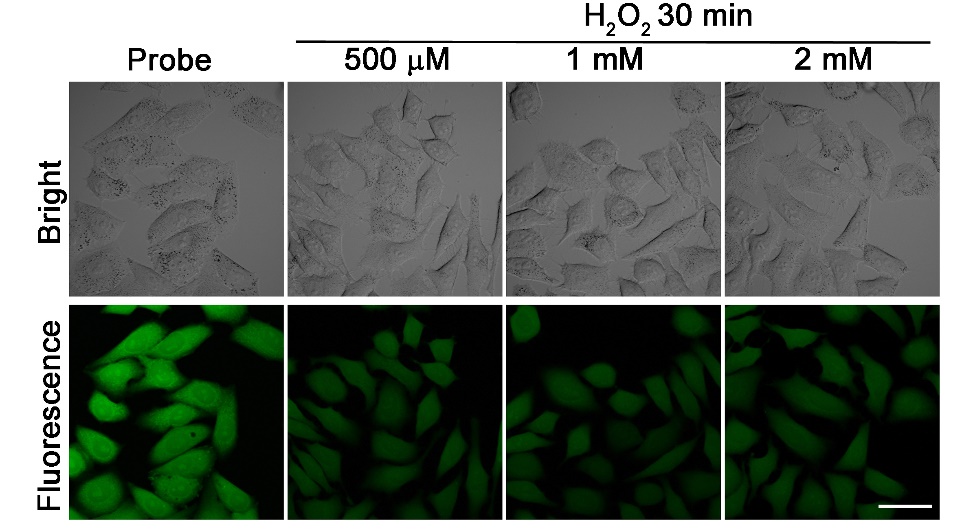
**

**Figure S4** (a) The cells incubated with probe (10 μM) for 5 min. (b-d) The cells pre-treated with H_2_O_2_ for 30 min and then incubated with probe (10 μM) for 5 min. Scale bar: 40 μm.

**4. Synthesis**

**

**

**Scheme S1** Synthetic routes of compounds 1-6.

**4.1 Synthesis of Compound 1**

Compounds 1a-6a was obtained according to previous methods.[[1-5](#_ENREF_1)]

General synthetic procedures: Compound 1a-6a (1.0 mmol) was dissolved in DCM (20 mL), and m-CPBA (516 mg, 3.0-5.0 mmol) was added successively to the solution. The mixture was stirred overnight at room temperature. Upon solvent evaporation, the crude product was purified by silica gel column chromatography.

**Compound 1**: ^1^H NMR (500 MHz, CDCl_3_) δ 8.39 – 8.25 (m, 1H), 8.15 (d, *J* = 8.1 Hz, 1H), 8.13 – 8.08 (m, 1H), 7.98 (d, *J* = 7.7 Hz, 1H), 7.63 – 7.52 (m, 1H), 7.51 – 7.39 (m, 1H), 3.14 (s, 2H). ^13^C NMR (126 MHz, CDCl_3_) δ 165.27, 154.01, 142.08, 138.42, 135.31, 128.27, 128.16, 126.83, 126.09, 123.81, 121.82, 44.49.

**Compound 2**: ^1^H NMR (500 MHz, CDCl_3_) δ 8.55 (d, *J* = 8.4 Hz, 1H), 8.40 (d, *J* = 16.4 Hz, 1H), 8.25 (d, *J* = 8.4 Hz, 1H), 8.17 (d, *J* = 8.9 Hz, 1H), 8.14 – 7.99 (m, 2H), 7.85 – 7.76 (m, 1H), 7.72 (dd, *J* = 11.1, 4.0 Hz, 1H), 3.09 (s, 2H), 2.17 (s, 3H), 1.73 (t, *J* = 7.4 Hz, 2H). ^13^C NMR (126 MHz, CDCl_3_) δ 182.13, 150.09, 143.35, 139.79, 138.34, 137.50, 134.09, 132.13, 131.90, 130.40, 128.91, 128.14, 127.99, 127.25, 122.94, 115.73, 112.26, 54.62, 45.30, 44.31, 26.50, 14.97. HRMS (m/z): 404.1685.

**Compound 3**: ^1^H NMR (500 MHz, CDCl_3_) δ 9.11 (dd, *J* = 8.7, 0.9 Hz, 1H), 8.83 – 8.67 (m, 2H), 8.57 (d, *J* = 7.7 Hz, 1H), 7.99 (dd, *J* = 8.6, 7.3 Hz, 1H), 4.27 – 4.03 (m, 2H), 1.83 – 1.68 (m, 2H), 1.56 – 1.29 (m, 2H), 0.99 (t, *J* = 7.4 Hz, 3H). ^13^C NMR (126 MHz, CDCl_3_) δ 163.32, 162.75, 140.66, 131.91, 129.92, 129.75, 129.65, 129.36, 128.93, 127.69, 127.45, 123.59, 44.73, 40.60, 30.06, 20.31, 13.79.

**Compound 4**: ^1^H NMR (500 MHz, CDCl_3_) δ 8.20 (dd, *J* = 7.9, 1.6 Hz, 1H), 8.00 (d, *J* = 8.4 Hz, 2H), 7.78 (d, *J* = 8.4 Hz, 2H), 7.72 (ddd, *J* = 8.6, 7.2, 1.7 Hz, 1H), 7.65 (d, *J* = 16.0 Hz, 1H), 7.55 (d, *J* = 8.2 Hz, 1H), 7.46 – 7.38 (m, 1H), 6.93 (d, *J* = 16.0 Hz, 1H), 6.40 (s, 1H), 3.10 (s, 3H). ^13^C NMR (126 MHz, CDCl_3_) δ 178.34, 160.46, 155.90, 140.86, 140.20, 134.29, 134.05, 128.23, 128.09, 125.74, 125.26, 124.03, 117.85, 111.97, 44.46. HRMS (m/z) [M + H]^+^: 327.0693.

**Compound 5**: ^1^H NMR (500 MHz, CDCl_3_) δ 8.64 (d, *J* = 15.9 Hz, 1H), 8.21 (dd, *J* = 7.9, 1.6 Hz, 1H), 8.17 (dd, *J* = 7.9, 1.2 Hz, 1H), 7.81 (d, *J* = 7.8 Hz, 1H), 7.76 – 7.70 (m, 2H), 7.60 (dd, *J* = 12.1, 4.6 Hz, 2H), 7.46 – 7.40 (m, 1H), 3.11 (s, 4H). ^13^C NMR (126 MHz, CDCl_3_) δ 178.51, 160.48, 155.96, 138.38, 135.12, 134.13, 134.12, 132.26, 129.75, 129.59, 128.44, 125.85, 125.64, 125.32, 124.00, 118.25, 112.03, 44.57. HRMS (m/z) [M + H]^+^: 327.0691.

**Compound 6**: ^1^H NMR (500 MHz, CDCl_3_) δ 7.99 (d, *J* = 9.2 Hz, 1H), 6.72 (s, 1H), 6.66 (dd, *J* = 9.3, 2.6 Hz, 1H), 6.57 (d, *J* = 2.6 Hz, 1H), 3.45 (q, *J* = 7.1 Hz, 4H), 3.19 (s, 3H), 1.24 (t, *J* = 7.1 Hz, 6H). ^13^C NMR (126 MHz, CDCl_3_) δ 160.13, 157.23, 151.38, 151.11, 126.23, 110.83, 109.61, 101.90, 98.08, 44.92, 43.64, 12.39. HRMS (m/z) [M + H]^+^: 296.0974.

**4.2 NMR spectra of Compound 1-6**

**
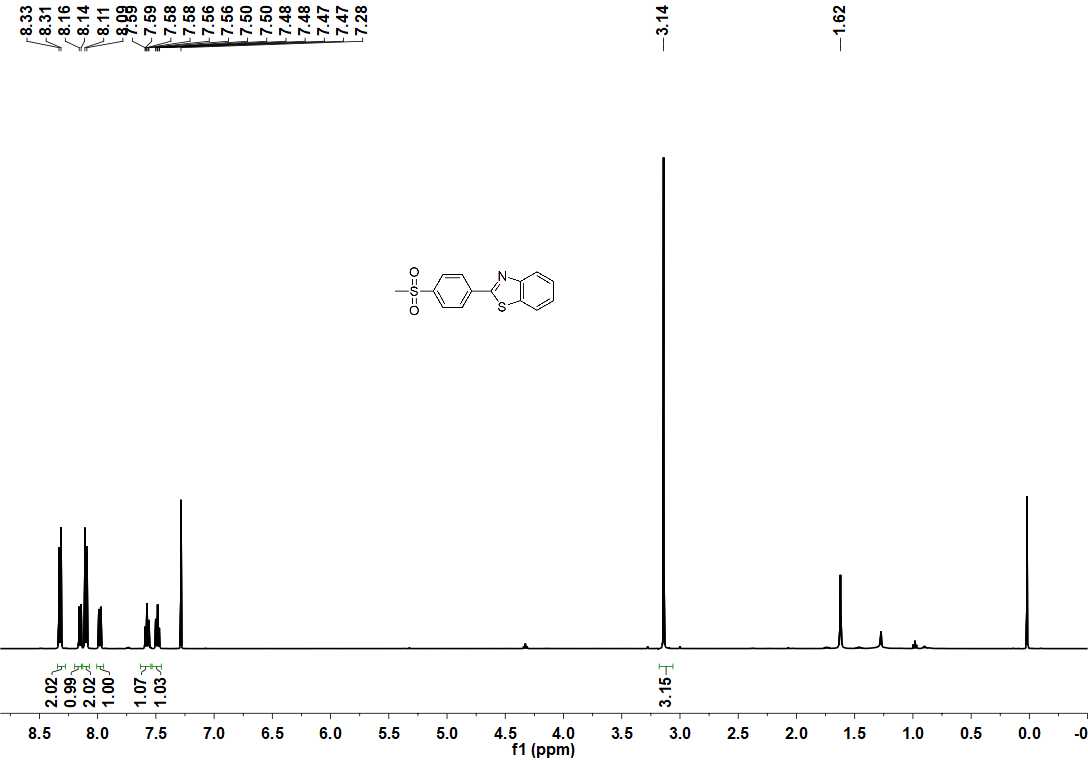
**

**
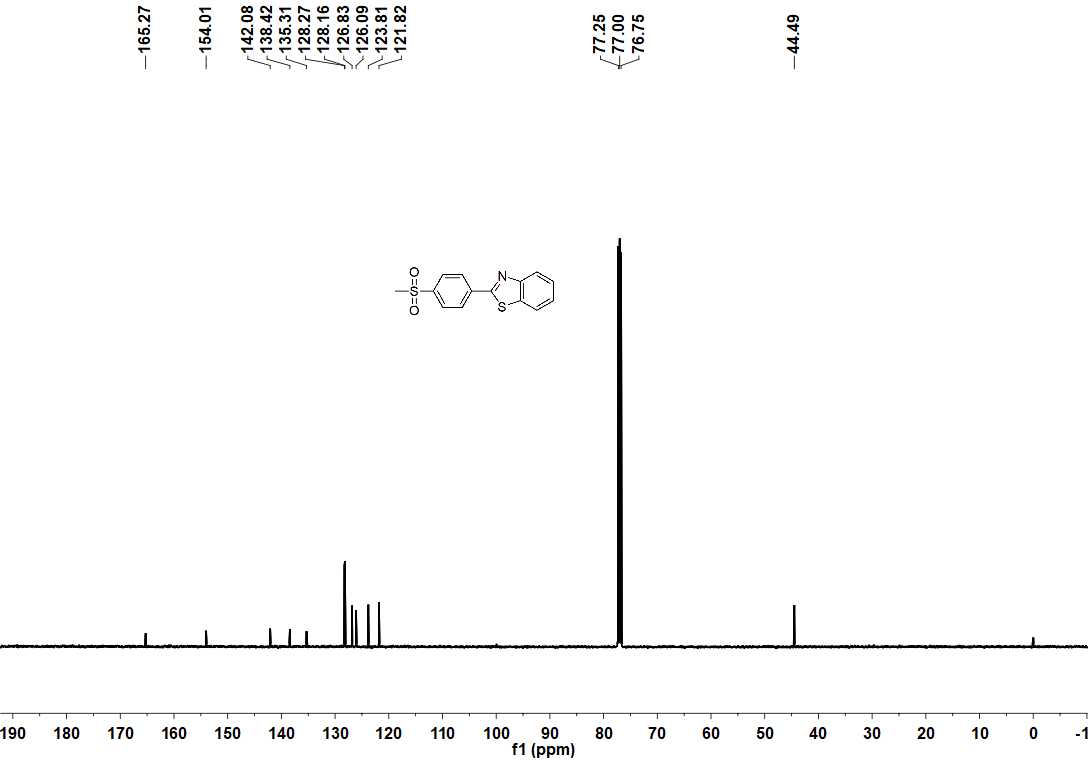
**

**
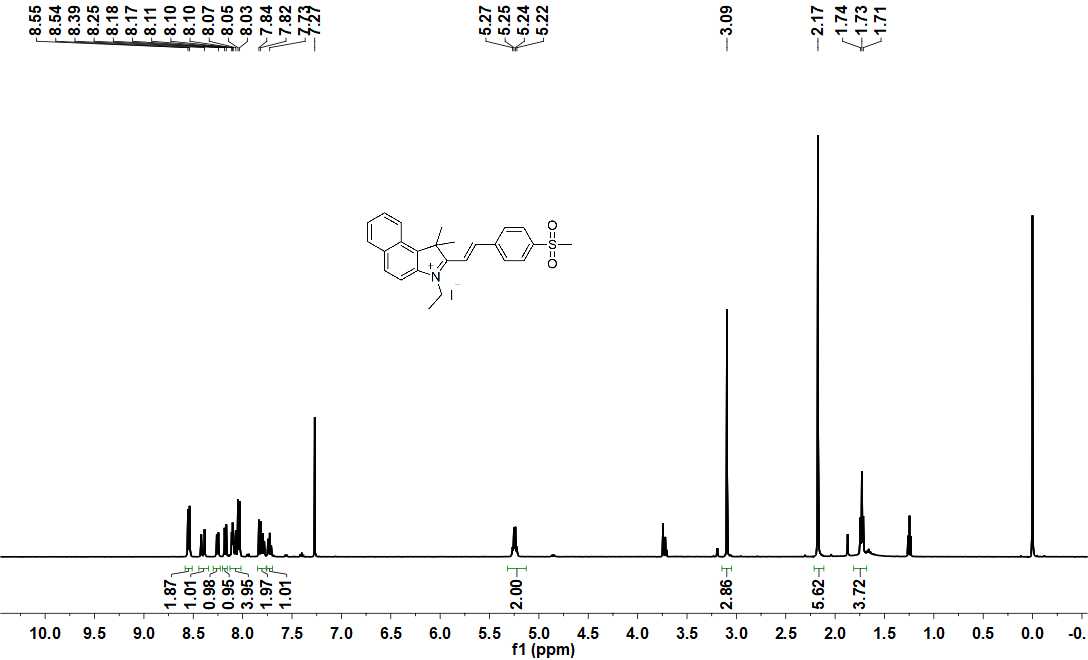
**

**
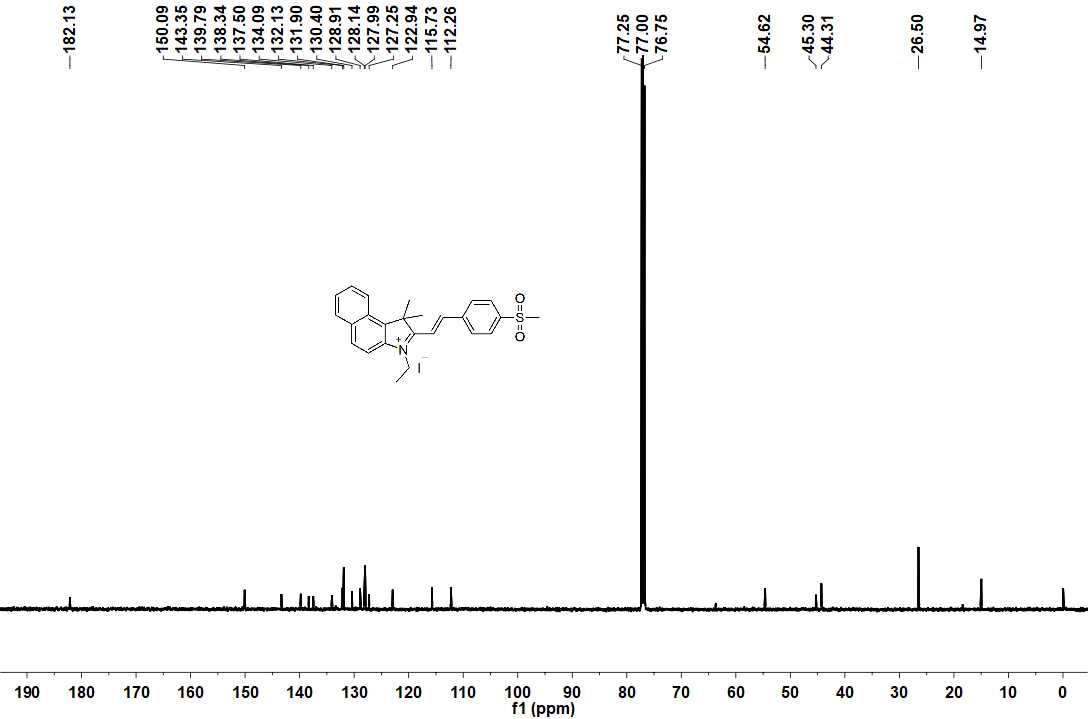
**


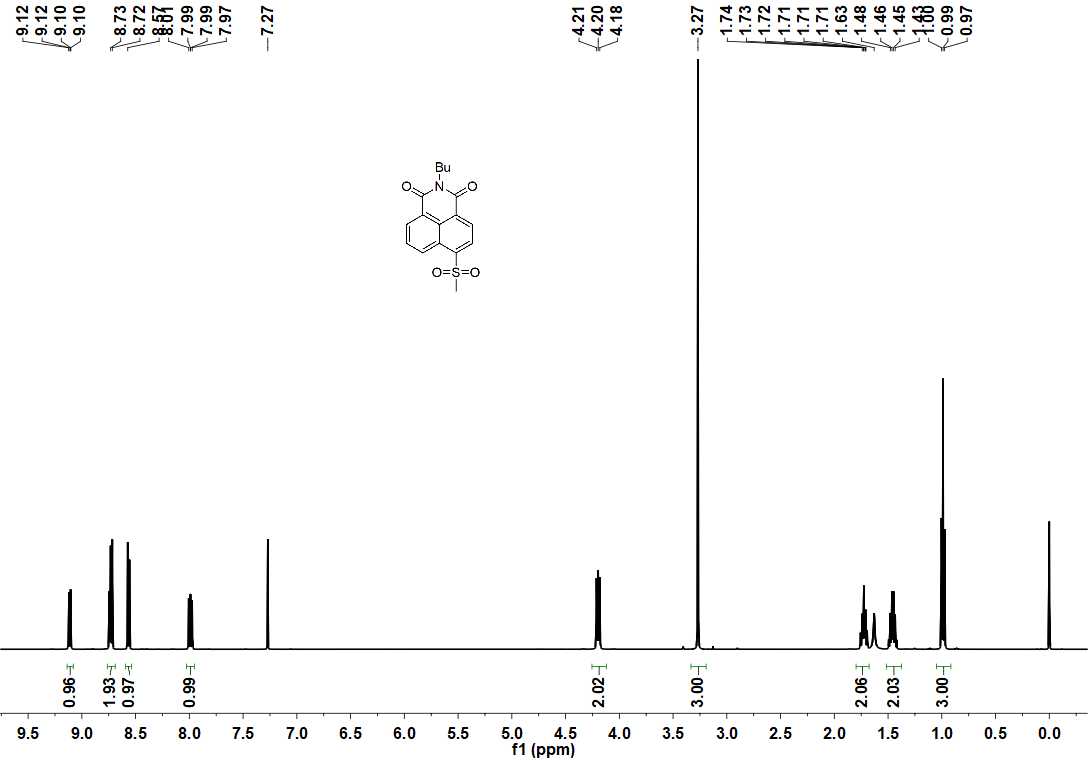


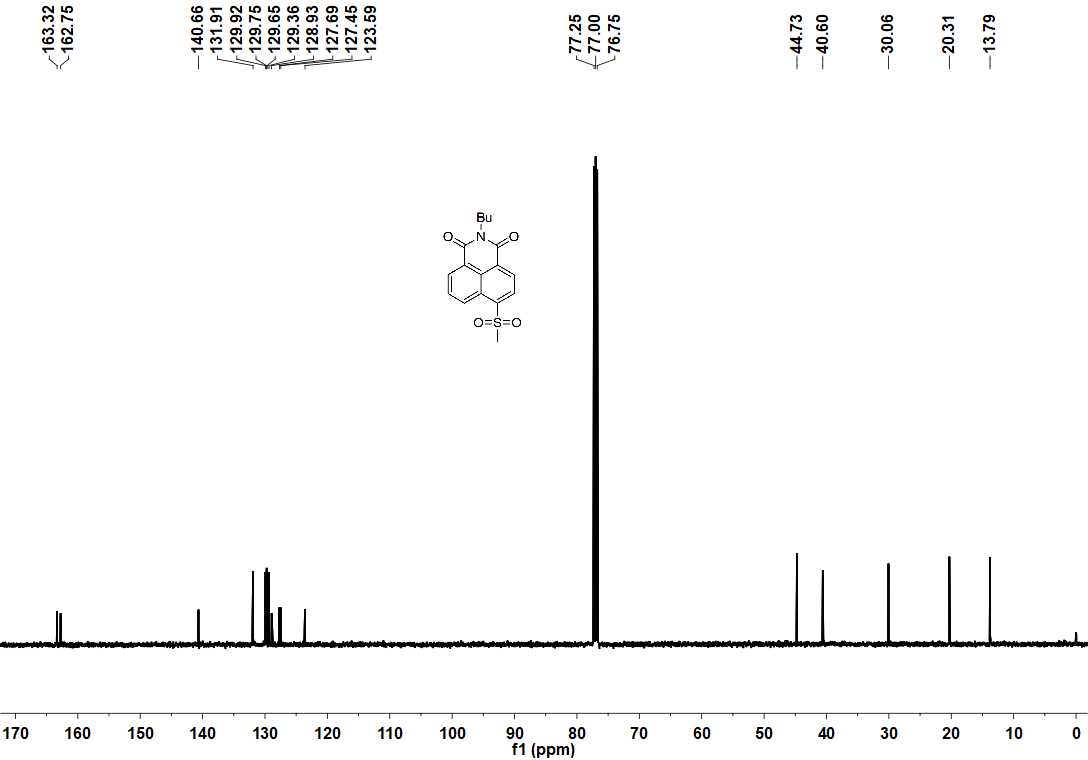


**
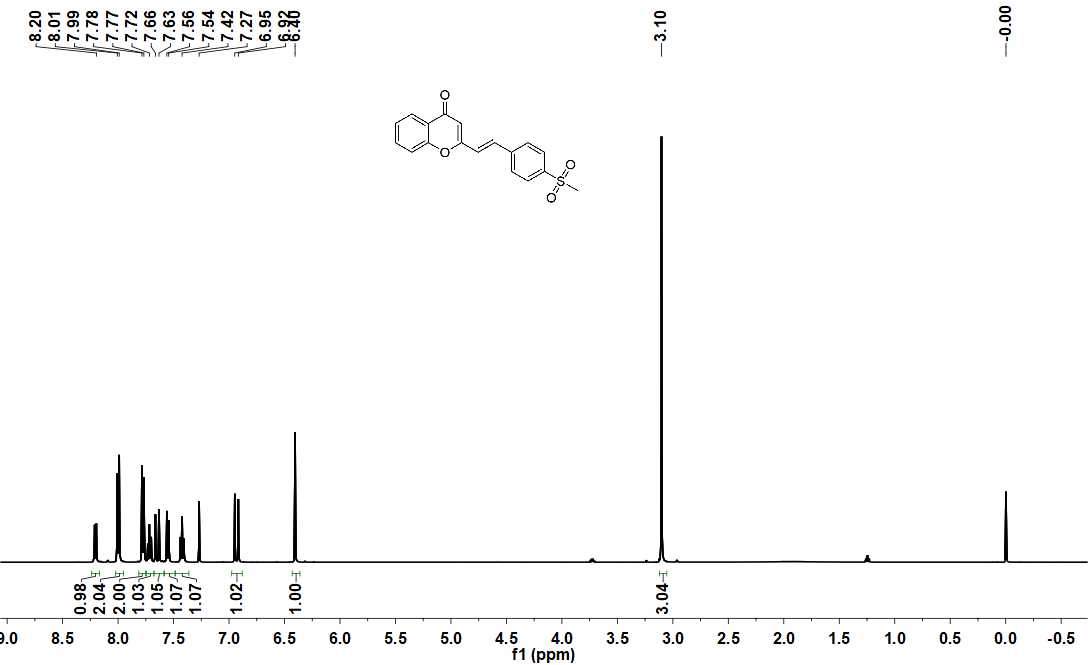
**

**
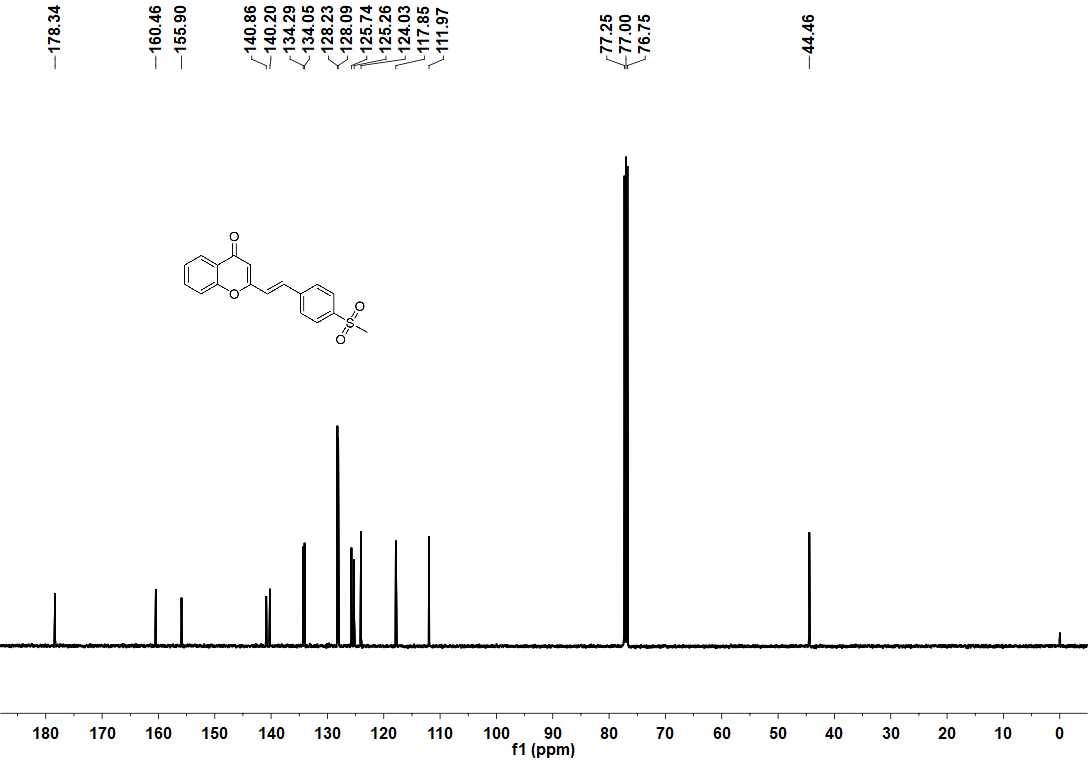
**


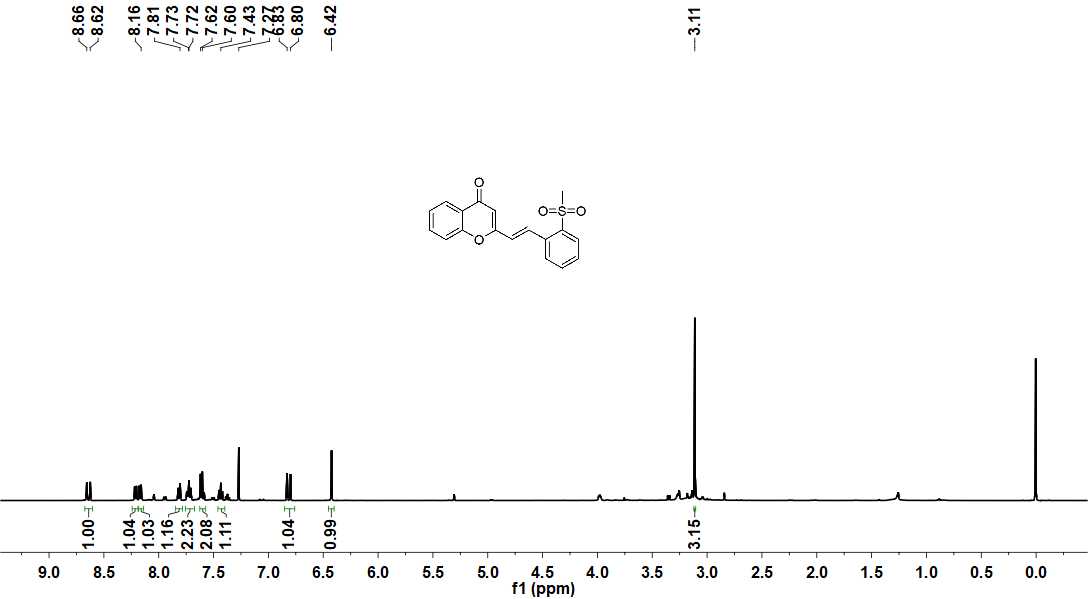


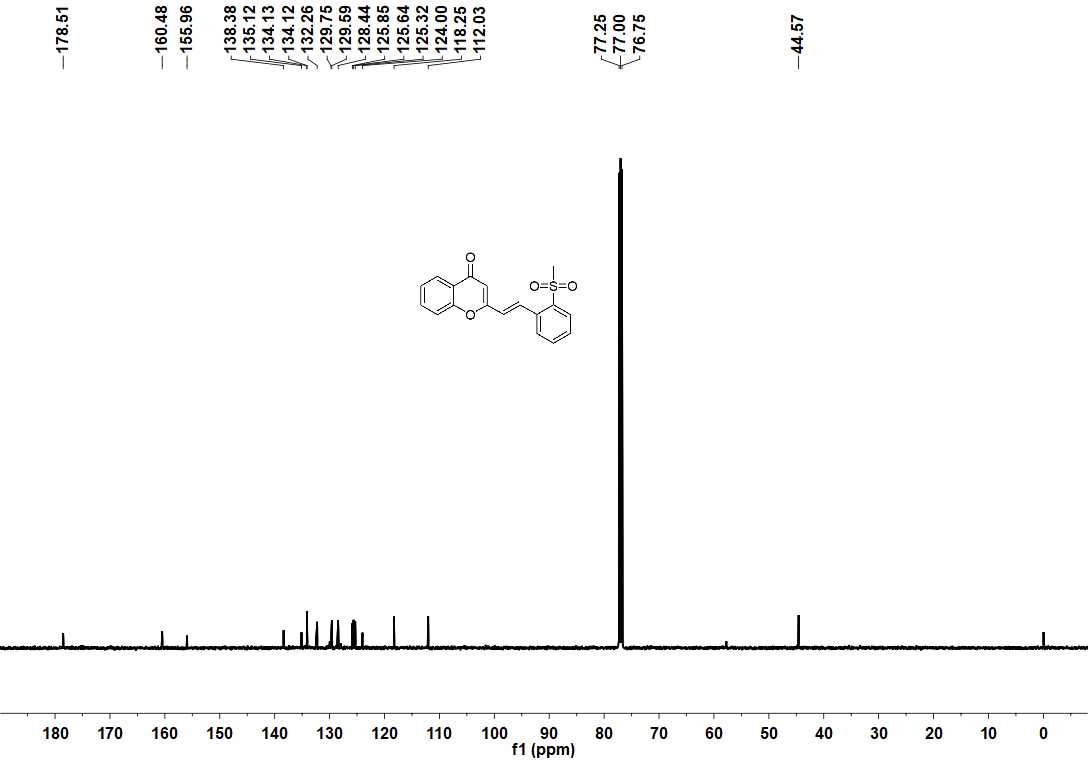


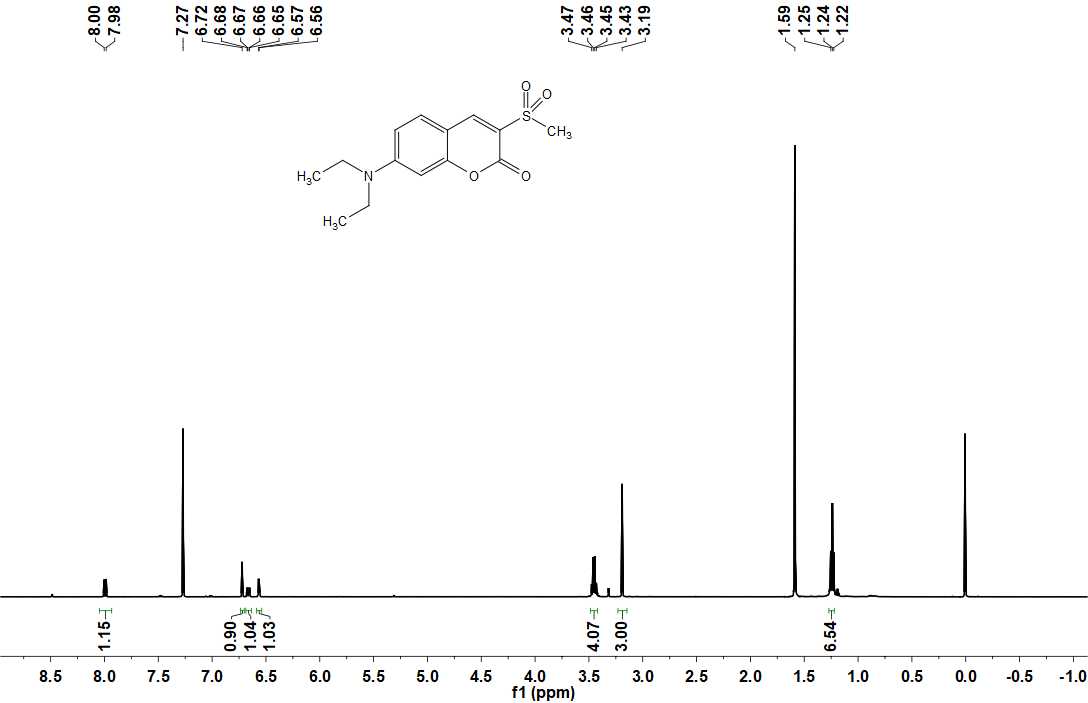


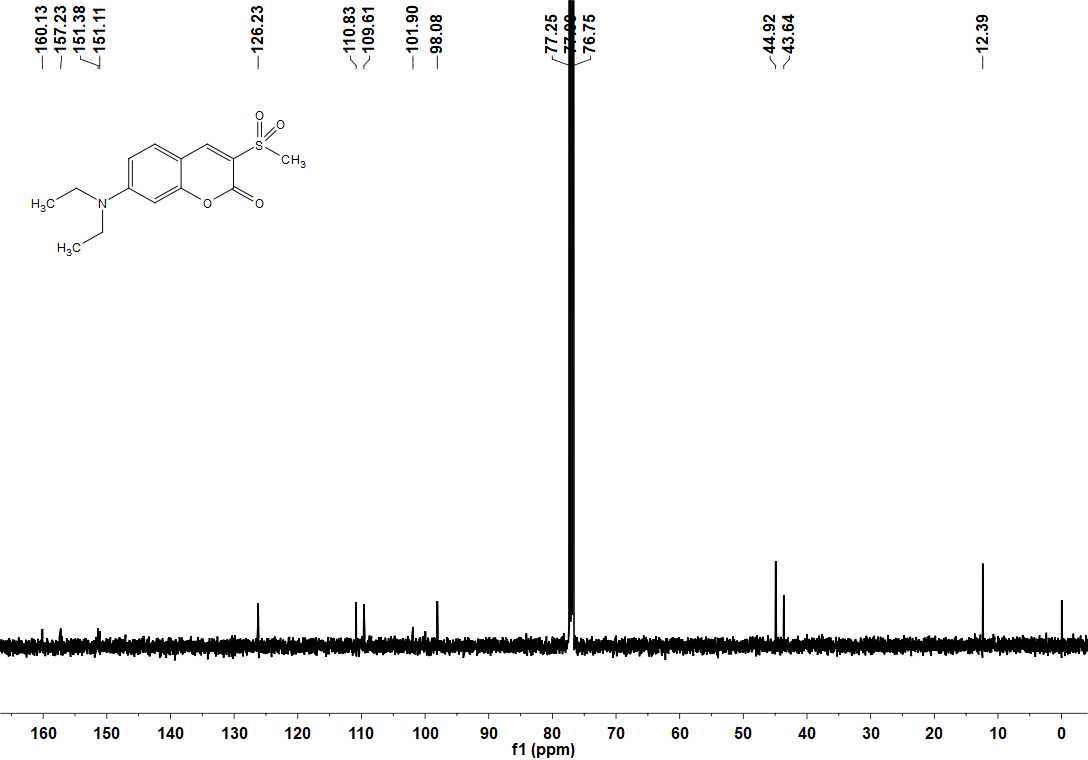


**References**

[1] Liu S, Feng D, Zhang L, et al. A reaction-based ratiometric fluorescent probe for mercury ion detection in aqueous solution. *Spectrochimica Acta Part A: Molecular and Biomolecular Spectroscopy*, 2020, 243(118817

[2] Zhang X, Zhang L, Liu S, et al. Insight into sulfur dioxide and its derivatives metabolism in living system with visualized evidences via ultra-sensitive fluorescent probe. *Journal of hazardous materials*, 2022, 423(127179

[3] Chen R, Lu H, Liu C, et al. Novel chemosensors for detection of glutathione by reduction or substitution of naphthalimide derivatives containing sulfoxide or sulfone substituents. *Bioorganic & medicinal chemistry letters*, 2015, 25(1): 59-61

[4] Zhang L, Peng S, Sun J, et al. A ratiometric fluorescent probe of methionine sulfoxide reductase with an improved response rate and emission wavelength. *Chemical communications*, 2019, 55(10): 1502-1505

[5] Zhang LW, Kang J, Liu SD, et al. A chemical covalent tactic for bio-thiol sensing and protein labeling agent design. *Chemical communications*, 2020, 56(77): 11485-11488
